# Additional Serine/Threonine Phosphorylation Reduces Binding Affinity but Preserves Interface Topography of Substrate Proteins to the c-Cbl TKB Domain

**DOI:** 10.1371/journal.pone.0012819

**Published:** 2010-09-22

**Authors:** Qingxiang Sun, Rebecca A. Jackson, Cherlyn Ng, Graeme R. Guy, J. Sivaraman

**Affiliations:** 1 Department of Biological Sciences, National University of Singapore, Singapore, Singapore; 2 Institute of Molecular and Cell Biology, Biopolis, Singapore, Singapore; Griffith University, Australia

## Abstract

The E3-ubiquitin ligase, c-Cbl, is a multi-functional scaffolding protein that plays a pivotal role in controlling cell phenotype. As part of the ubiquitination and downregulation process, c-Cbl recognizes targets, such as tyrosine kinases and the Sprouty proteins, by binding to a conserved (NX/R)pY(S/T)XXP motif via its uniquely embedded SH2 domain (TKB domain). We previously outlined the mode of binding between the TKB domain and various substrate peptide motifs, including epidermal growth factor receptor (EGFR) and Sprouty2 (Spry2), and demonstrated that an intrapetidyl hydrogen bond forms between the (pY-1) arginine or (pY-2) asparagine and the phosphorylated tyrosine, which is crucial for binding. Recent reports demonstrated that, under certain types of stimulation, the serine/threonine residues at the pY+1 and/or pY+2 positions within this recognition motif of EGFR and Sprouty2 may be endogenously phosphorylated. Using structural and binding studies, we sought to determine whether this additional phosphorylation could affect the binding of the TKB domain to these peptides and consequently, whether the type of stimulation can dictate the degree to which substrates bind to c-Cbl. Here, we show that additional phosphorylation significantly reduces the binding affinity between the TKB domain and its target proteins, EGFR and Sprouty2, as compared to peptides bearing a single tyrosine phosphorylation. The crystal structure indicates that this is accomplished with minimal changes to the essential intrapeptidyl bond and that the reduced strength of the interaction is due to the charge repulsion between c-Cbl and the additional phosphate group. This obvious reduction in binding affinity, however, indicates that Cbl's interactions with its TKB-centered binding partners may be more favorable in the absence of Ser/Thr phosphorylation, which is stimulation and context specific *in vivo*. These results demonstrate the importance of understanding the environment in which certain residues are phosphorylated, and the necessity of including this in structural investigations.

## Introduction

The epidermal growth factor receptor (EGFR) is a 170 kDa transmembrane glycoprotein and, similar to a number of other tyrosine kinases, it has an important role in the regulation of a wide range of cellular functions. EGFR is activated by specific ligands, including EGF and transforming growth factor α (TGF- α), which stimulates receptor dimerization and the onset of intrinsic tyrosine kinase activity [1], [2], [3]. This in turn results in the autophosphorylation of multiple residues within the intracellular region [4], a mechanism common to various tyrosine kinases, leading to downstream signaling and changes to cell phenotype. Activating mutations, receptor overexpression and other modes of signal amplification from EGFR can prohibit the proper attenuation of the signal, and have been linked to causing a range of cancers, particularly lung cancer [5], [6], [7]. As as result, EGFR regulation has become the focus of many research laboratories. Given that the kinase can undergo rapid autophosphorylation, it is imperative that a mechanism is in place to strictly control receptor signaling; one key mechanism is through c-Cbl-mediated ubiquitination and degradation.

The protein product of the Casitas B-lineage lymphoma (c-Cbl) gene is a multi-domain, multi-functional scaffolding protein with E3-ubiquitin ligase activity that is crucial for regulating cell signaling pathways. c-Cbl regulates cell proliferation, differentiation and morphology in response to growth factors, hormones and cytokines [8], [9], binding to substrates via its tyrosine-kinase binding motif (TKB) in its N-terminus. Through a cascade of steps, c-Cbl then induces the ligation of ubiquitin molecules to its substrates, targeting them for internalization and degradation. Elucidating the TKB recognition sequence has been central to understanding the biochemistry of tyrosine kinase downregulation by Cbl proteins. Until recently, the c-Cbl TKB domain was thought to have an uncharacteristically flexible binding mode, utilizing three disparate motifs – (D/N)XpY(S/T)XXP or RpY(S/T)XXP or DpYR – to accomplish its task [10], [11], [12], [13]. The TKB domain is essentially an embedded SH2 domain, and experimental precedent had indicated that SH2 domains employ a relatively rigid recognition strategy for binding target proteins, which contrasted with the supporting Cbl literature. This led us to investigate how Cbl achieved such flexibility in binding, and whether there was a hitherto unknown mechanism common to all three motifs. We reported the crystal structures of the c-Cbl TKB domain in complex with peptides derived from Sprouty2, Sprouty4, EGFR, Syk tyrosine kinase and c-Met receptor [14], and found that the c-Cbl TKB domain indeed employed a unique mechanism for binding that was almost identical across the different substrates. The c-Met motif, DpYR, interacted with Cbl in the reverse orientation, and an obligatory intrapeptidyl bond formed between the phosphorylated tyrosine and the (pY-1)Arg (EGFR and reversed c-Met) or the (pY-2)Asn (Sprouty2, Sprouty4 and Syk). This bond was essential for orientating the peptide into the positively-charged binding pocket on Cbl, to permit binding. In the absence of the residue in the pY-1 or -2 positions, respectively, binding was abolished.

In examining Cbl's interaction with target proteins, we and others did not examine the degree to which phosphorylation of an adjacent Ser or Thr residue at the pY+1 position in the consensus sequence would impact on the binding affinity of c-Cbl with its targets. While there is an abundance of evidence both *in vitro* and *in vivo* to show that phosphorylation of Tyr1069 on EGFR is crucial for c-Cbl binding and receptor ubiquitination, it is less-well characterized how phosphorylation of the additional serine residues within the target sequence affects substrate recognition by c-Cbl. Recent work has revealed that both Ser1070 and Ser1071, immediately adjacent to the Tyr1069 are critical for EGFR desensitization, internalization and degradation [15]. Furthermore, these two serine residues can be phosphorylated by a number of ligands [16], including EGF [17], light-activated Calphostin-C [18], hydrogen peroxide [19], TAK1 kinase [20], p38 mitogen-activated protein kinase (MAPK) [21] and UV irradiation [22], and their phosphorylation leads to receptor desensitization [17], internalization [18] and is necessary to inhibit its own kinase activity [23]. Recently, it was reported that Sprouty2 is also endogenously phosphorylated on the threonine residue (Thr56) adjacent to the phosphorylated tyrosine in the pY+1 position in growing culture cells [24]. Similar to EGFR, Sprouty2 binds to the c-Cbl TKB domain with high affinity via its phosphorylated c-Cbl binding motif centred on Tyr55. Indeed, we and others have shown that it binds to and sequesters c-Cbl from EGFR through this same tyrosine residue, thereby enhancing EGFR expression levels on the cell surface [25], [26], [27], [28]. In our previous study, we demonstrated this sequestration model to be feasible, with Sprouty2 showing a higher binding affinity to c-Cbl than EGFR [14].

As phosphorylation can cause large structural changes to binding, we sought to investigate the effect of multiple phosphorylation on the binding between c-Cbl and EGFR or Sprouty2, in continuation with our earlier work [14]. Furthermore, we wanted to verify whether this additional phosphorylation would change the binding affinity of c-Cbl with Sprouty2 and EGFR. Any variation in binding or the total abrogation of binding, as a result of this adjacent Ser/Thr phosphorylation, may indicate that a stimulation-specific interaction exists between c-Cbl and its particular substrate.

We employed doubly and triply phosphorylated peptides – pYpT-Spry2, pYpS-EGFR and pYpSpS-EGFR (hereafter simplified to ppSpry2, ppEGFR and tpEGFR) – and investigated changes in binding affinity with the TKB domain of c-Cbl. We also determined the crystal structure of the doubly phosphorylated EGFR and Spry2 peptides complexed with c-Cbl TKB at 2.2 and 2.1 Å resolutions, respectively, and observed that the phosphate group of the Ser/Thr residue shifts away from the c-Cbl phosphotyrosine binding pocket with minor conformational changes of the peptides in the interacting region. Using Surface Plasmon Resonance (SPR) studies, we observed that multiple phosphorylations significantly reduce the binding affinity of the peptides to the c-Cbl TKB, with the triply phosphorylated peptide having the lowest affinity amongst all the peptides tested. These results indicate that, while additional phosphorylation sites will affect the binding affinity of the peptides to c-Cbl, the overall conformation of the interaction does not change significantly. These modifications to the binding affinity suggest that in order to obtain accurate binding affinity data in structural investigations truly reflective of the *in vivo* setting, the type and duration of stimulation and the effect this stimulation has on the proteins of interest needs to be fully understood.

## Methods

### Surface plasmon resonance (SPR)

**Figure 1:**
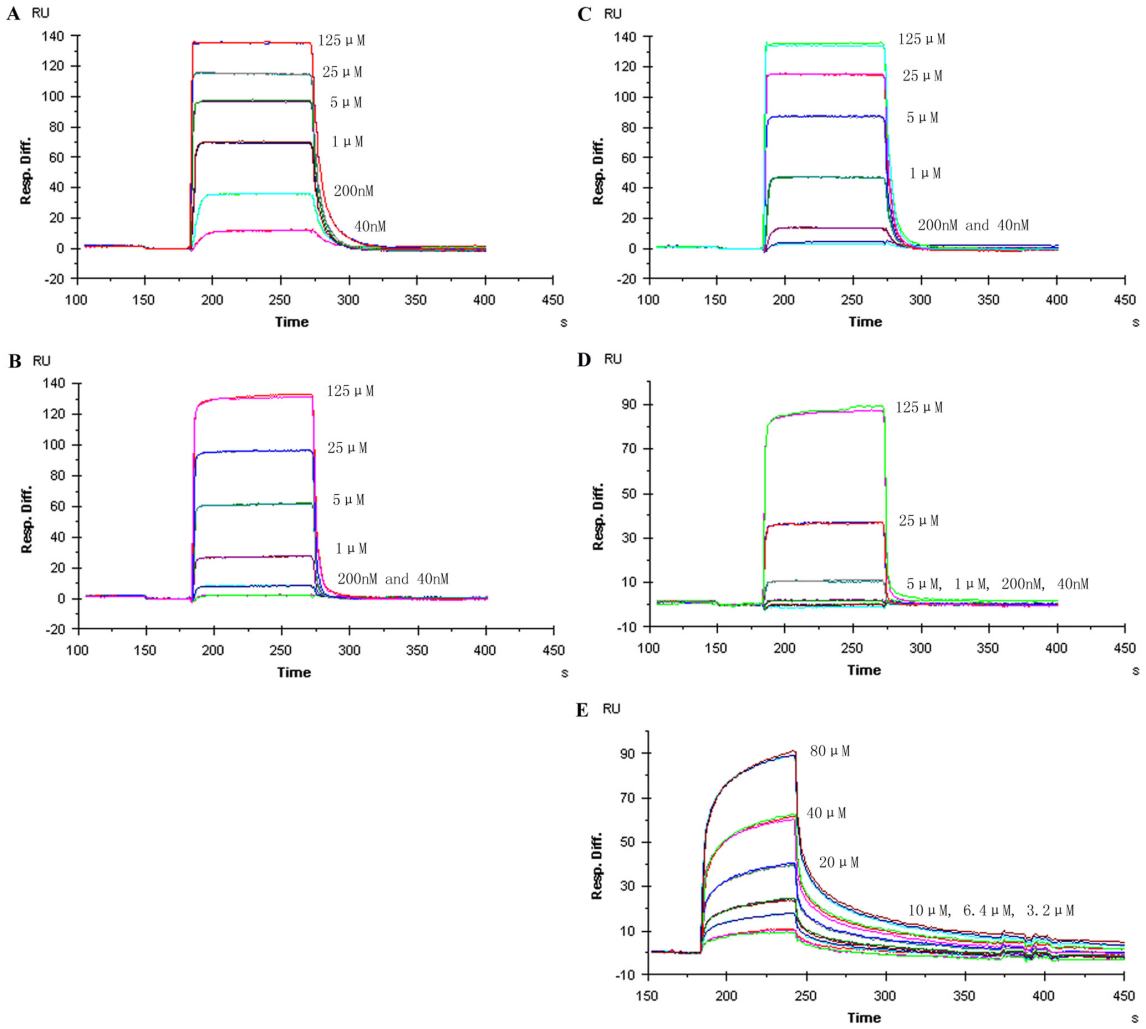
SPR kinetic analysis of pSpry2(A), ppSpry2(B), pEGFR(C), ppEGFR(D), tpEGFR(E) binding to c-Cbl TKB domain. Six peptide concentrations ranging from 40 nM to 125 µM are used for each peptide in this study. Each concentration is repeated two times and the raw data is shown. The y axis shows the response unit (RU) differences (minus the reference) and the x axis shows the time (s). The kinetic values are listed in Table 1.

SPR experiments were performed with a Biacore 3000 (Biacore AB, Uppsala, Sweden). c-Cbl protein (50 ng/µl, 110 µl, in 5 mM sodium citrate pH 6.5) was immobilized onto a CM5 Chip as per the manufacturer's recommendations. The running buffer consisted of 20 mM Na Hepes, pH 7.0 and 150 mM NaCl. Experiments were performed at 25°C. Different concentrations of peptides in running buffer were applied to the chip surface at a flow rate of 20 µl/min (Figure 1). Regeneration using running buffer at 50 µl/min for 1 min resulted in a stable baseline corresponding to the starting baseline level. c-Cbl TKB, that was immobilized on the reference cells, was inactivated by a 1 min flow of 10 mM H_2_SO_4_ through the cells. The resulting c-Cbl TKB lost its ability to bind to the phosphorylate peptides and was used as a control to eliminate false positive bindings, as well as to confirm complete coverage of nonspecific binding sites. All sensorgrams were processed using automatic correction for non-specific bulk refractive index effects. The equilibrium constant (KD) was determined by the 1∶1 Langmuir binding fitting model provided by the Biacore 3000 instrument software.

### Complex formation, crystallization and data collection

Cloning, expression and purification of c-Cbl was performed as described previously [14]. Multiply phosphorylated peptides of human Sprouty2^49–61^ (IRNTNEpYpTEGPT) and EGFR^1063–1075^ (DSFLQRpYpSSDPT, DSFLQRpYpSpSDPT) were purchased from GL Biochem, reconstituted in c-Cbl-TKB storage buffer, incubated with purified c-Cbl-TKB in two- to five-fold molar excess, and concentrated to 5 mg/ml using Amicon Ultra ultrafiltration devices (Millipore, Billerica, MA). ppSpry2:TKB complex crystallized using PACT screen solution (condition number 57: 20% PEG 3350 and 0.2 M K/Na tartrate) by mixing 1 µl of reservoir solution with 1.5 µl of protein (hanging drop) at room temperature. The ppEGFR:TKB complex crystallization condition was initially identified from PACT screen and further optimized. The highest quality crystals of ppEGFR:TKB complex were obtained when 1 µl of reservoir solution containing 20% (w/v) PEG 3350, 150 mM NaK tartrate, 0.1 M Bis-Tris propane pH 6.1 was mixed with 1.5 µl of protein (hanging drop) at room temperature. For both complexes, diffraction quality crystals were obtained in 3 days. The mother liquor was supplemented with 20% glycerol for cryo-protection. X-ray diffraction data was collected in-house with a Bruker X-8 PROTEUM system.

### Structure solution and refinement

Diffraction data was integrated and scaled using HKL2000 [29]. Both complex structures were solved using molecular replacement method with the program MolRep [30] using the previously determined coordinates of the c-Cbl TKB domain as a search model [14] (pdb code: 3BUO). There was one complex molecule in the asymmetric unit. The resulting model with the electron density map was examined in the program COOT [31], and necessary manual model building was performed. Several alternating cycles of map fitting and refinement using the program Refmac5 [32], led to the convergence of R-values 0.179 (R_free_ = 0.224) for ppSpry2:TKB and 0.188 (R_free_ = 0.221) for ppEGFR:TKB.

## Results

### Additional phosphorylation reduces peptide binding affinity

Phosphorylation is an important covalent modification that precisely controls the outcome of many *in vivo* functions, and there is evidence to suggest that multiple sites of phosphorylation may be present within the c-Cbl binding motif of EGFR and Spry2, depending on the type of stimulation to which the cells are exposed. The Ser/Thr residue at the pY+1 position is partially conserved in a sequence alignment of c-Cbl binding peptides and, while the phosphorylation of the serine residues Ser1070 and Ser1071 are essential for the regulation of EGFR expression [15], the importance of phosphorylation of the pY+1 Thr56 on Spry2 is less-well understood [24]. Furthermore, it is currently unknown how a change in the phosphorylation of these adjacent Ser/Thr residues affects the topographical interaction of substrates with c-Cbl.

Previously, we identified that the Tyr55 phosphorylated Spry2 peptide (hereafter referred to as pSpry2) showed the highest binding affinity to the c-Cbl TKB domain as compared to the other peptides studied, including the Tyr1069 phosphorylated EGFR peptide [14] (hereafter referred to as pEGFR). We therefore employed Surface Plasmon Resonance (SPR) to assess the relative binding affinities of the TKB domain with the singly phosphorylated pEGFR and pSpry2 and the multiply phosphorylated peptides, pTyr55/pThr56 Spry2 (ppSpry2), pTyr1069/pSer1070 EGFR (ppEGFR) and pTyr1069/pSer1070/pSer1071 EGFR (tpEGFR) (Table 1).

**Table 1:** Binding parameters for the c-Cbl:peptide interactions.

Peptide	Sequence	k_on_	k_off_	K_d_	χ^2^
		(_M_^−1^s^−1^)	(s^−1^)	(µM)	
pSpry2	IRNTNEpYTEGPT	3.7×10^5^	0.139	0.31	4.85
ppSpry2	IRNTNEpYpTEGPT	7.7×10^4^	0.295	3.83	6.68
pEGFR	DSFLQRpYSSDPT	2.8×10^5^	0.279	1.00	0.68
ppEGFR	DSFLQRpYpSSDPT	6.5×10^3^	0.167	25.8	0.09
tpEGFR	DSFLQRpYpSpSDPT	687	0.022	31.5	3.36

χ^2^ is the statistical error between the experimental and theoretical models. χ^2^ is defined as Σ (R_f_-R_x_)^2^/(n-p), where R_f_ is the fitted value at a given point, R_x_ is the experimental value at the same point, n is the number of data points, and p is the number of fitted parameters.

As compared to pSpry2, the double phosphorylation on Spry2 caused a 12-fold reduction in binding affinity (0.31 µM to 3.83 µM; Fig. 1A and B). Similarly, the additional phosphorylation on EGFR significantly reduced the binding affinity of EGFR to Cbl when compared with the singly phosphorylated EGFR peptide, with 26- and 31-fold decreases for ppEGFR and tpEGFR, respectively (Fig. 1C–E). Notably, the difference between the double and triple phosphorylation for EGFR was not considerable, suggesting that phosphorylation at the pY+1 position is predominantly responsible for the decrease in binding affinity. Interestingly, the k_on_ and k_off_ are significantly different between the doubly and triply phosphorylated peptides, with tpEGFR showing an approximately 10 times slower k_on_ and k_off_.

The phosphotyrosine interaction site in the c–Cbl TKB domain is basic, and therefore, we expected that the addition of phosphate group(s) on Ser1070 and Ser1071 would have enhanced the interaction between EGFR and c-Cbl. The same would be predicted for Spry2′s interaction with c-Cbl. However, we observed the opposite, with additional phosphorylation significantly reducing the binding affinity of Cbl with EGFR and Spry2. In order to understand the structural basis for the reduced binding affinities, we crystallized the complexes of the c-Cbl TKB domain with the doubly phosphorylated peptides of EGFR and Spry2. As the triply phosphorylated peptide did not significantly reduce the binding affinity over the doubly phosphorylated peptide, we did not investigate this interaction further.

### Double phosphorylation is unfavorable for binding and weakens the interaction

The c-Cbl TKB domain consists of three tightly-connected domains: a divergent SH2 domain that binds to the phosphorylated tyrosine; a four-helix bundle (4H) which packs against the SH2 domain and completes the phosphotyrosine binding pocket; and a calcium-binding EF-hand, which wedges between the SH2 and 4H domains [10]. The close contact between these three domains is maintained by several hydrogen bonds and an extensive network of hydrophobic interactions. Consistent with other studies, our previous study demonstrated that the pSpry2 and pEGFR peptides interact with c-Cbl TKB through (1) a positively-charged pocket into which the phosphorylated tyrosine inserts and establishes the majority of the hydrogen bonds, and (2) a hydrophobic cluster, which forms significant interactions with the conserved C-terminal proline residue of the bound peptide.

**Figure 2:**
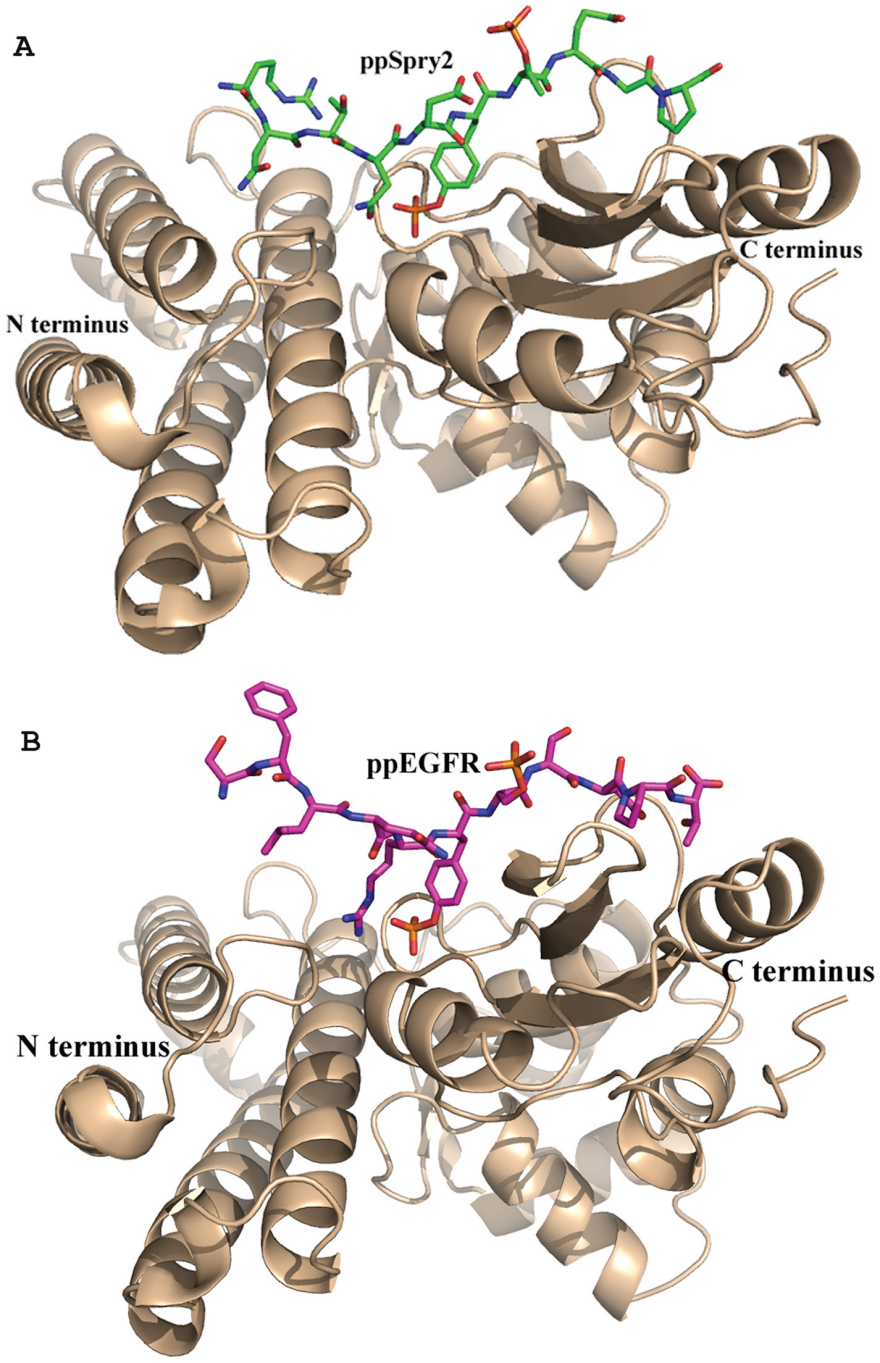
Crystal structure of TKB:ppSpry2 and TKB:ppEGFR complexes. (**A**) Ribbon diagram of the TKB:ppSpry2 and (**B**) TKB:ppEGFR, N- and C- termini are labeled. c-Cbl-TKB is in gold. ppSpry2 (green) and ppEGFR (magenta) peptides are shown in stick represenations. These figures and the following figures in this manuscript were prepared using the program PyMol [40].

The structures of ppSpry2:TKB and ppEGFR:TKB co-complexes were determined to 2.2 and 2.1 Å resolution, respectively (Table 2), with one complex molecule in the asymmetric unit of both complexes (Fig. 2). The c-Cbl TKB domains are similar in both complexes (0.77 Å rmsd for 2427 atoms) and, aside from minor side chain differences, conformation of the TKB domain is similar to those complexes that we and others previously described using the single phosphopeptide [14].

**Table 2:** Crystallographic data and refinement statistics.

Data set	ppSpry2-Cbl	ppEGFR-Cbl
**Data collection** *		
Cell parameters (Å)	122.09, 122.09, 55.69	122.77, 122.77, 55.43
Space group	P6	P6
Resolution range (Å)	50–2.2 (2.28–2.20)	50–2.06 (2.13–2.06)
Wavelength (Å)	1.5418	1.5418
Observed reflections	217204	377642
Unique reflections	24185	29542
Completeness (%)	99.8 (99.5)	99.7 (98.6)
Overall *I/σI*	16.9 (3.47)	16.6(2.64)
R_sym_ a (%)	0.089 (0.436)	0.115(0.399)
Solvent content (%)	61%	60%
**Refinement statistics**		
Resolution range (Å)	50–2.2	20–2.1
R_work_ b (no. of reflections)	0.179 (21715)	0.188 (25131)
R_free_ c (no. of reflections)	0.224 (1235)	0.221 (1425)
RMSD bond lengths (Å)	0.026	0.024
RMSD bond angles (deg)	2.118	1.760
No. of protein atoms/ligand atoms/water molecules	2435/91/162	2435/100/314
**B-factors (Å^2^)**		
Average B-factors of protein atoms	25.38	31.42
rms B-factor of protein atoms	2.02	1.85
Average B-factor of ligand atoms	31.48	39.70
rms B-factor of ligand atoms	3.06	1.89
**Ramachandran plot**		
Most favored regions (%)	97.3	98.0
Generously allowed regions (%)	2.0	2.0
Disallowed regions (%)	0.7	0.0

a R_sym_  =  Σ|I_i_ -<I>|/Σ|I_i_| where I_i_ is the intensity of the i^th^ measurement, and <I> is the mean intensity for that reflection.

b R_work_  =  Σ| F_obs_ - F_calc_|/Σ|F_obs_| where F_calc_ and F_obs_ are the calculated and observed structure factor amplitudes, respectively.

c R_free_  =  as for R_work_, but for 5.0% of the total reflections chosen at random and omitted from refinement for all datasets.

*The highest resolution bin statistics were given in the parenthesis.

**Figure 3:**
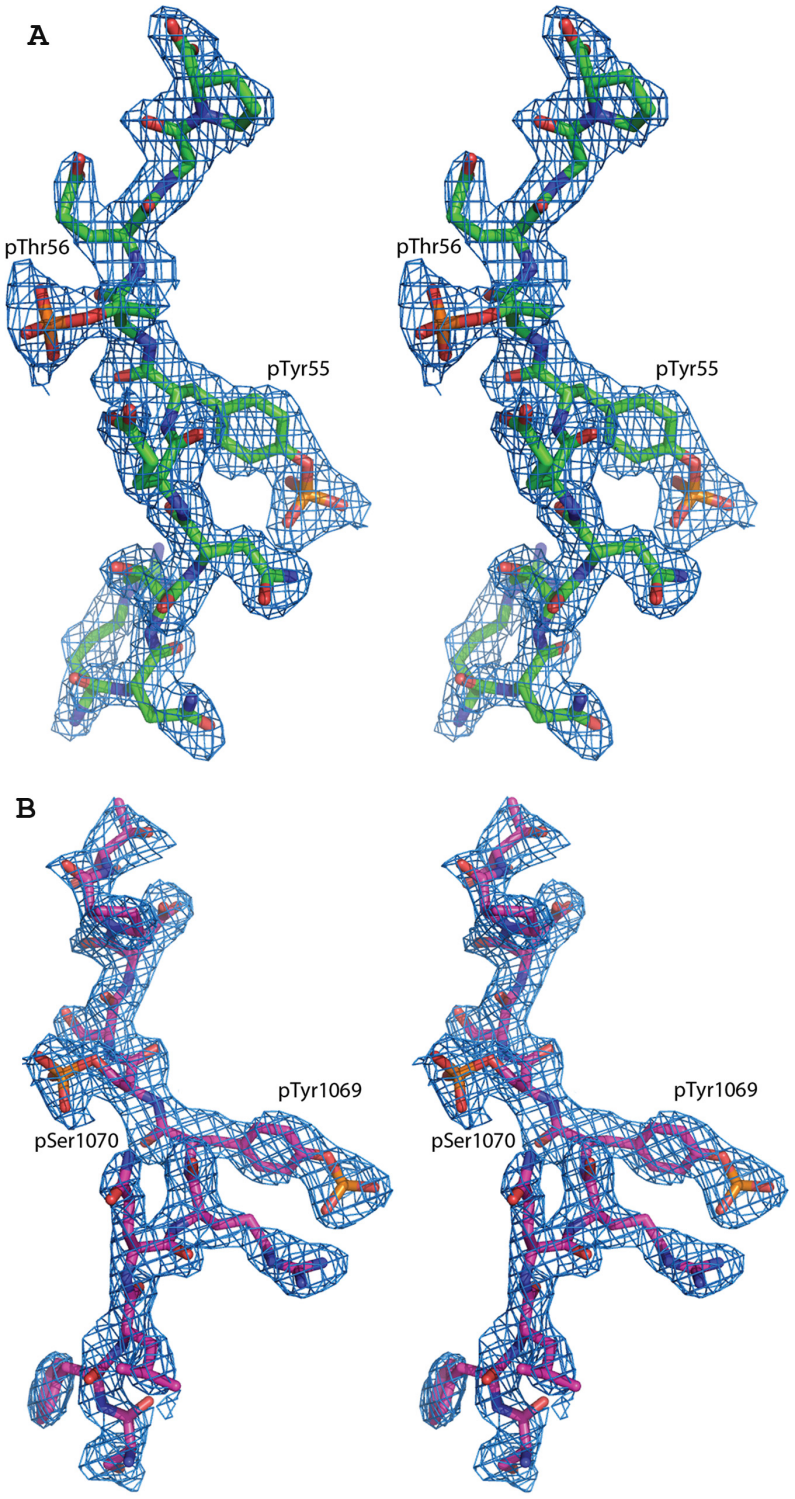
Stereo-view of the 2*Fo-Fc* simulated annealing omit map of peptides from (A) ppSpry2-Cbl and (B) ppEGFR-Cbl. All atoms within 3.5 Å of the peptides were omitted prior to refinement. Maps were contoured at a level of 1.0σ.

**Figure 4:**
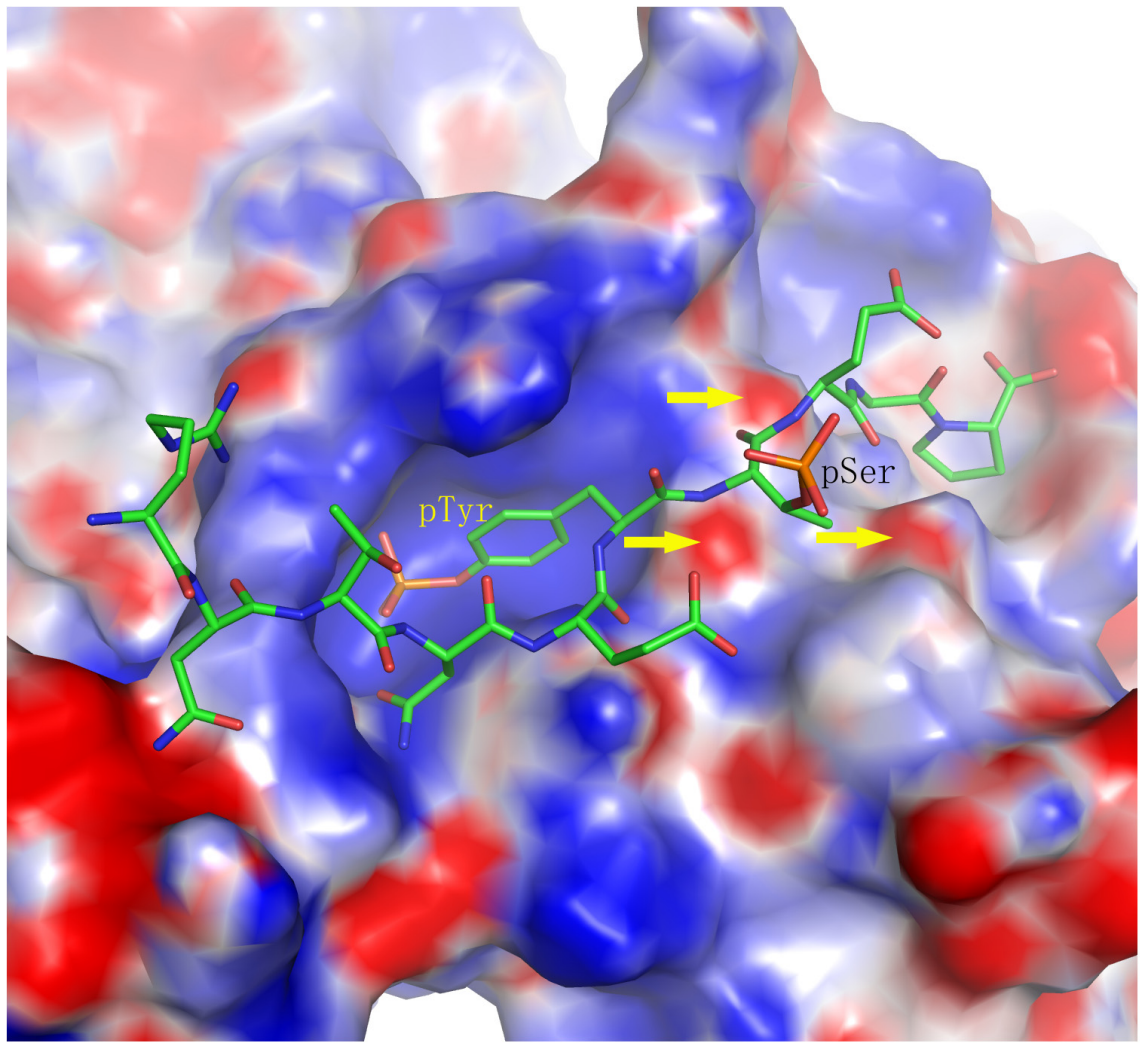
The electrostatic surface potential at the region where the phosphorylated peptide binds to the c-Cbl TKB domain. ppSpry2 peptide is shown in stick representation. The yellow arrows show the negatively-charged region on c-Cbl that electrostatically repels the phosphate group of the phosphorylated serine (pSer) residue.

**Figure 5:**
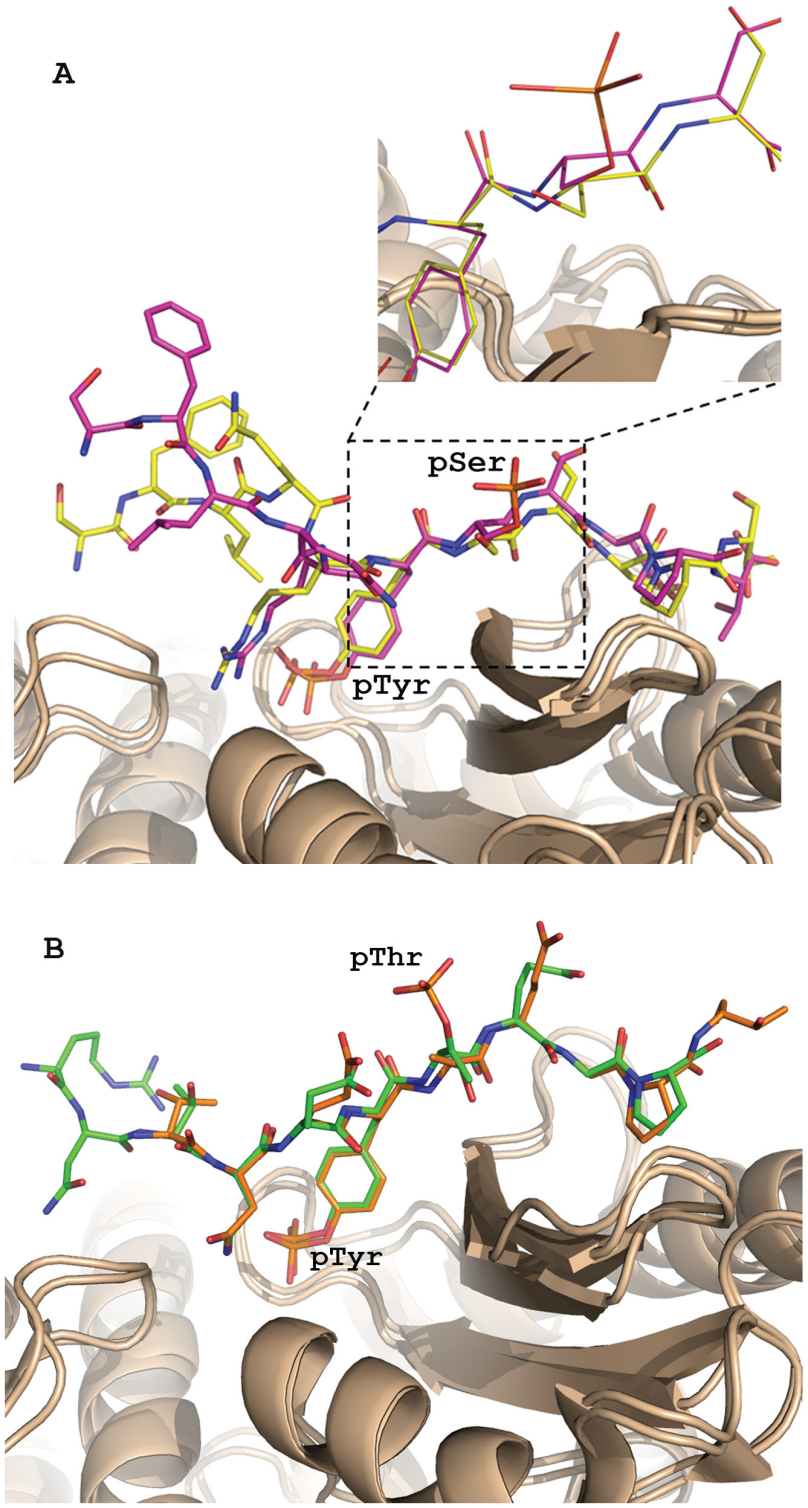
Superposition of TKB:peptide complexes. Peptides are shown in colored stick representation, and c-Cbl-TKB is shown in ribbon representation (gold). (**A**) ppEGFR (purple) versus pEGFR (yellow). The rmsd between the superimposed TKB SH2 domains from both complexes is 0.351 Å for 81 Cα atoms. The enlarged view shows the peptide shift. The shift of the backbone atoms of the phosphorylated residues at the Y+1, Y+2 and Y+3 positions is approximately 0.7 to 1 Å, while the shift of the phosphorylated tyrosine backbone atom is about 0.1 Å. (**B**) ppSpry2 (green) versus pSpry2 (orange). The SH2 domain (81 Cα atoms) of the TKB from both complexes was superimposed with an rmsd of 0.219 Å.

The phosphate groups of the phosphorylated Thr56 of Spry2 and the phosphorylated Ser1070 of EGFR are clearly visible in the electron density maps (Fig. 3). Surprisingly, unlike the phosphate group of the tyrosine residue, which makes six hydrogen bonding contacts with c-Cbl, the phosphate groups of Thr56 and Ser1070 have no interaction with c-Cbl and are both shifted away from the surface (Fig. 2). Given this lack of interaction between the second phosphate group and c-Cbl TKB domain, it would be expected that the affinity between c-Cbl and ppEGFR or ppSpry2 would be unaffected. However, the SPR results show an obvious reduction in binding affinity for the doubly phosphorylated peptides when compared with the singly phosphorylated peptides. We noted no significant differences in the number of hydrogen bond contacts between c-Cbl and EGFR peptides (Table S1). However, the orientation of second phosphate group away from the surface of Cbl suggests that this phosphate group is not favorable. When we analyzed the electrostatic surface potential (Fig. 4), the results showed that the region on c-Cbl contacting the pY+1 and pY+2 positions of the peptides is negatively charged, which could repel the high negative charge on the phosphorylated serine or threonine residues. When the SH2 domains from the singly and doubly phosphorylated EGFR:TKB structures are superimposed, it is clear that the backbone atoms of the phosphoSer1070 and Ser1071 residues from ppEGFR are slightly pushed back from c-Cbl surface (largest displacement 1 Å) as compared to pEGFR (estimated coordinate error is 0.24 for ppEGFR and 0.44 for pEGFR by Luzzati plot) (Fig. 5A). This is in contrast to the backbone Cα of the pTyr, for which there is a negligible shift when the two complexes are superimposed. These observations clearly demonstrate electrostatic repulsion is occurring due to the additional phosphorylation.

Interestingly, the other residues in the middle of the peptide remained unchanged (Arg1068 and pTyr1069), and only small changes were observed in the C-terminal residues (Arg1068 – Thr1074) between the pEGFR:TKB and the ppEGFR:TKB complexes. However, a significant conformational change was observed at the N-terminus of ppEGFR, with residues Ser1064 – Gln1067 also shifting away from c-Cbl TKB surface. The electron density map is weak at the N-terminal regions of both complexes; therefore this change is unlikely due to the additional phosphorylation of Ser1070 at the C-terminus.

The peptides in the pSpry2:TKB and ppSpry2:TKB complex structures adopted similar conformations (Fig. 5B), with comparable overall interactions. This is in agreement with the smaller reduction in binding affinity for between ppSpry2 and pSpry2 measured by SPR, as compared with the greater difference detected between pEGFR and both ppEGFR and tpEGFR. However, we did observe that the interaction of the Thr56 side chain of ppSrpy2 with Gln316, which was present in the pSpry2:TKB complex from our previous study [14], is absent in the ppSpry2 complex due to re-orientation of the side chain following phosphorylation. The electron density map for ppSpry2 shows well-defined N-terminal residues, with a contribution from Asn51 at the N-terminus of ppSpry2 that was not seen in the singly phosphorylated complex [14].

The buried surface areas and dissociation energy barriers of singly and doubly phosphorylated peptides were analyzed by PISA [33]. For the purposes of comparison, the same number of residues from c-Cbl (55–351), Spry2 (52–59) and EGFR (1064–1074) were used for analysis. The buried surface area for c-Cbl with pSpry2, ppSpry2, pEGFR or ppEGFR were 1041.6Å^2^, 1025.0Å^2^, 1331.9Å^2^ and 1261.3Å^2^, respectively (Table 3), which shows small differences in the buried area for singly and doubly phosphorylated peptides (1.6% and 5.4% reduction, respectively). However, the predicted dissociation energy barriers for the doubly phosphorylated peptide complexes were significantly lower (Table 3), indicating that these peptides have a lower affinity for the TKB domain than the singly phosphorylated peptides. It is worth mentioning here that the SPR results show that the reduced affinity is caused by the reduced association rate (k_on_), rather than a faster dissociation (k_off_).

**Table 3:** Comparison of interaction surface and dissociation energy barrier predicted with PISA [33].

Peptide complexed	Buried surface area	Dissociation barrier
with TKB	(Å^2^)	(kcal/mol)
pSpry2	1041.6	7.3
ppSpry2	1021.9	4.2
pEGFR	1331.9	6.7
ppEGFR	1268.6	4.0

Overall, the orientation of second phosphate group, the electrostatic surface environment on c-Cbl, the backbone shifts at the Y+1 and Y+2 positions, and the lowered dissociation energy barriers suggest that the presence of a phosphate group at pY+1 position is unfavorable for binding, and weakens the affinity between the peptide and c-Cbl without abolishing the binding between the two proteins.

## Discussion

The aim of this paper was to address the important question of whether TKB-directed binding to c-Cbl would be disrupted by the additional serine/threonine phosphorylation that has been reported to occur endogenously for EGFR and Sprouty2 under various stimulated conditions. Studies have shown that EGFR can become phosphorylated on Ser1070 and Ser1071 by a range of stimuli, and recent work has demonstrated the potential for Thr56 of Spry2 to become phosphorylated in cultured cells. In both cases, these residues fall within the c-Cbl TKB binding motif, for which the consensus currently contains only a single phosphorylation on the tyrosine residue (Tyr1069 and Tyr55, respectively). As a first step toward understanding what may occur *in vivo*, we wanted to ascertain whether phosphorylation of the adjacent serine/threonine residue(s) would significantly alter peptide binding to the c-Cbl TKB domain. Further, we sought to determine whether this additional phosphorylation could significantly alter the binding affinity of proteins to c-Cbl. Our results suggest that while the binding topography does not significantly change with the additional phosphorylation, the binding affinity is considerably reduced by 12- and 30-fold for ppSpry2 and ppEGFR, respectively. This reduction appears to be primarily due to phosphorylation at pY+1 position, which is pushed away from the c-Cbl TKB surface by the phosphate group (Fig. 4). The side chain of the phosphorylated serine/threonine in ppSpry2 and ppEGFR is exposed towards the solvent, and thus it might not be involved in any structural changes.

Oksvold *et al.* addressed the function of the serine residues at 1046 and 1047 in EGFR (equivalent to Ser1070/1071 in the current numbering) via the use of multiple point mutations. They observed that by substituting these serine residues with alanine, EGF was unable to induce internalization and ubiquitination of the EGFR, even though the Y1045 (Y1069) was still able to be phosphorylated by EGF, and the c-Cbl-EGFR interaction was maintained [15]. These findings show that the serine residues are not necessary for the c-Cbl-EGFR interaction, which supports our earlier work revealing that only the (pY-2)Asp or (pY-1)Arg and the essential pTyr within the TKB binding motif are required to initiate substrate binding to c-Cbl [14]. The work by Oksvold and colleagues, however, also suggests that these two adjacent serines are responsible for directing EGFR internalization and ubiquitination. This disrupted internalization of serine-mutated EGFR has also been described elsewhere [34]. Both of these studies utilized amino acid substitution methods to test the importance of the serine residues; the substitution of these serine residues for alanine is not the same as dephosphorylating them and, as the serine side chain is sterically larger than an alanine side chain, it is plausible that some structural changes may have also contributed to the inhibited EGFR internalization. This aside, since binding to Cbl itself is unchanged, it may be assumed that these serine residues, whether phosphorylated or not, may be required to bind to functional complexes of the endocytotic and degradation machinery involved with receptor internalization, including the Cbl interacting protein of 85 kDa (CIN85) and endophilins, which bind to the tail end of the receptor during endocytosis [35].

One of c-Cbl's primary functions in the cell is to target and suppress the activity of phosphorylated tyrosines on proteins, particularly tyrosine kinases, through the process of ubiquitination. However, this process does not directly correlate to c-Cbl binding. c-Cbl identifies candidate ubiquitination substrates by way of its TKB domain, and then directs the transfer of ubiquitin molecules from ubiquitin conjugating (E2) enzymes to these targeted substrates by virtue of its RING domain, lying adjacent to the TKB domain (reviewed in [36]). In this regard, the function of the TKB domain is simple: to identify phosphorylated tyrosine residues on activated substrates harboring the TKB recognition sequence. However, the process of ubiquitination is complex, and not all of c-Cbl's targets are in fact also ubiquitination targets. For example, the APS (adapter with a plekstrin homology and Src homology-2 domains) protein, which binds to the TKB domain through a derivate of the (NX/R)pY(S/T)XXP sequence – RA(V/I)XNQpY(S/T) – is not a ubiquitination substrate. Instead, the APS protein employs c-Cbl as a docker protein in the insulin receptor complex [37]. While the TKB-binding sequence on APS harbors some additional residues N-terminal to the phosphotyrosine, it still retains the essential Asn residue in the pY-2 position, and forms the unique intrapeptidyl bond between this residue and the phosphorylated tyrosine [14]. Therefore, the manner in which APS binds to c-Cbl is identical to Cbl's other substrate targets, suggesting that binding *per se* has no direct consequence on the ubiquitination process.

Whilst EGFR and Sprouty2 still bind to c-Cbl TKB domain in the presence of additional serine phosphorylation, these binding affinities are weaker than we originally detected with the single tyrosine phosphorylated peptides [14], and may indicate a weak association with c-Cbl in cells. However, is not possible to accurately speculate what might be the physiological outcomes of these adjacent phosphorylated residues. A lesser affinity for the c-Cbl TKB domain is not necessarily directly correlated with less ubiquitination. Whilst there is a paucity of information on binding affinity versus ubiquitination, our unpublished observation is that binding affinity does not correlate with ubiquitination, and this is partly validated by the high affinity binding in some cases where ubiquitination is not the final outcome. For instance, we have measured that the APS protein binds the c-Cbl TKB domain with high affinity, however it is not ubiquitinated [14]. Similarly, pSprouty2, which has the highest binding affinity over all the singly phosphorylated peptides tested, is seemingly poorly degraded, with cell lysates displaying high expression levels of Sprouty2 even in the presence of overexpressed c-Cbl in stimulated conditions [14], [38]. The inverse is true for the c-Met receptor, which shows very weak interactions with c-Cbl in ITC experiments and in immunoprecipitations, but is very quickly degraded when c-Cbl is present in abundance [12], [14]. This discrepancy in binding affinity versus ubiquitination has been reviewed elsewhere [39]. What is likely to be more relevant for determining the degree of ubiquitination, is the binding affinity of the conjugated ubiquitin with the target substrate, the mode of ubiquitination (for example, mono- versus polyubiquitination), and the E2 or ubiquitin-conjugating enzyme that is employed. Whilst ubiquitination is unlikely to be directly associated with binding affinity, the combination of tyrosine and serine phosphorylation may together provide the optimum chance of ubiquitination through the complex recruitment of key machinery of the endocytic pathway.

### Protein Data Bank code

Coordinates were deposited with accession codes 3OB1 and 3OB2 for ppSpry2 and ppEGFR respectively.

## Supporting Information

Table S1 Hydrogen bond contacts between Spry2 and EGFR peptides and c-Cbl. These data are generated by ccp4 CONTACT program.
(0.00 MB PDF)
