# Supplementary material for: Additional Serine/Threonine Phosphorylation Reduces Binding Affinity but Preserves Interface Topography of Substrate Proteins to the c-Cbl TKB Domain
Source: PLoS One. 2010 Sep 22;5(9):e12819. doi: 10.1371/journal.pone.0012819 (PMC2943896; doi:10.1371/journal.pone.0012819)
Supplement: Table S1 — Hydrogen bond contacts between Spry2 and EGFR peptides and c-Cbl. These data are generated by ccp4 CONTACT program. (0.00 MB PDF) [file pone.0012819.s001.pdf]

pSpry2 ATOMS	c-Cbl ATOMS	DISTANCE
53(ASN) ND2	81(PRO) O	2.89
54(GLU) O	274(TYR) OH	2.67
55(PTR) OH	294(ARG) NH2	2.89
55(PTR) O2P	297(CYS) N	2.95
55(PTR) O3P	298(THR) N	2.87
	298(THR) OG1	2.58
	296(SER) OG	2.74
	297(ARG) N	3.32
56(THR) N	316(GLN) O	2.83
56(TPO) O	317(THR) OG1	2.51
56(THR) OH	316(GLN) O	3.25
58(GLY) N	317(THR) OG1	3.17

ppSpry2 ATOMS	c-Cbl ATOMS	DISTANCE
51(ASN) ND2	79(ASN) O	2.82
	86(ASP) OD2	2.78
53(ASN) OD1	81(PRO) O	2.98
54(GLU) O	274(TYR) OH	2.69
55(PTR) O2P	294(ARG) NH1	2.86
	297(CYS) N	3.06
55(PTR) O3P	296(SER) OG	2.66
	298(THR) N	2.89
	298(THR) OG1	2.75
55(PTR) OH	294(ARG) NH2	2.87
56(TPO) N	316(GLN) O	2.86
56(TPO) O	317(THR) OG1	2.83
58(GLY) N	317(THR) OG1	3.06

pEGFR ATOMS	c-Cbl ATOMS	DISTANCE
1068(ARG) O	274(TYR) OH	2.81
1068(ARG) NH1	81(PRO) N	3.17
	81(PRO) O	2.81
1068(ARG) NH2	81(PRO) O	2.66
1069(PTR) OH	294(ARG) NH2	2.88
1069(PTR) O2P	294(ARG) NH2	3.03
1069(PTR) O3P	298(THR) N	2.77
	298(THR) OG1	2.94
	296(SER) OG	3.13
1070(SER) N	316(GLN) O	2.75
1070(SER) O	317(THR) OG1	2.56
1072(ASP) OD2	322(LYS) NZ	3.06
1074(THR) OG1	334(GLU) OE1	2.96

ppEGFR ATOMS	c-Cbl ATOMS	DISTANCE
1067(GLN) NE2	314(ILE) O	3.11
1068(ARG) NH2	81(PRO) O	2.78
1068(ARG) O	274(TYR) OH	2.73
1069(PTR) O2P	294(ARG) NH2	3.15
	297(CYS) N	3.00
1069(PTR) O3P	296(SER) OG	2.67
	298(THR) N	2.81
	298(THR) OG1	2.53
1069(PTR) OH	294(ARG) NH2	2.67
1070(SEP) N	316(GLN) O	2.86
1070(SEP) O	317(THR) OG1	2.85
1074(THR) OG1	322(LYS) NZ	2.89
